# Effects of Horse Housing System on Energy Balance during Post-Exercise Recovery

**DOI:** 10.3390/ani9110976

**Published:** 2019-11-14

**Authors:** Malin Connysson, Marie Rhodin, Anna Jansson

**Affiliations:** 1 Wången National Center for Education in Trotting, Vången 110, S-835 93 Alsen, Sweden; 2 Department of Anatomy, Physiology and Biochemistry, Swedish University of Agricultural Sciences, SE-75007 Uppsala, Sweden; marie.rhodin@slu.se (M.R.);

**Keywords:** NEFA, Standardbred trotters, feed intake

## Abstract

**Simple Summary:**

Horse management aims to keep horses healthy and ensure good performance and animal welfare. Many horses are currently kept in individual box stalls indoors, a housing system that limits free movement, exploration, and social interaction, and may also subject horses to lower air quality. The alternative is a free-range housing system where horses are kept in groups outdoors. Anecdotal information indicates concerns among sports horse trainers that lack of rest in such systems delays recovery and impairs performance. This study examined whether recovery after competition-like exercise in Standardbred trotters was affected by housing system. The results showed that a free-range housing system did not delay recovery in Standardbred trotters, and in fact had positive effects on appetite and recovery of energy balance.

**Abstract:**

This study examined the effects of two housing systems (free-range and box stalls) on recovery of energy balance after competition-like exercise in Standardbred horses. Eight adult geldings (mean age 11 years) were used. The study had a change-over design, with the box stall (BOX) and free-range group housing (FreeR) treatments each run for 21 days. The horses were fed forage ad libitum and performed two similar race-like exercise tests (ET), on day 7 and day 14 in each treatment. Forage intake was recorded during the last 6–7 days in each period. Blood samples were collected before, during, and until 44 h after ET. Voluntary forage intake (measured in groups with four horses in each group) was higher in FreeR horses than BOX horses (FreeR: 48, BOX: 39, standard error of the mean (SEM) 1.7 kg (*p* = 0.003)). Plasma non-esterified fatty acids (NEFA) was lower at 20–44 h of recovery than before in FreeR horses (*p* = 0.022), but not in BOX horses. Housing did not affect exercise heart rate, plasma lactate, plasma urea, or total plasma protein concentration. Thus the free-range housing system hastened recovery in Standardbred trotters, contradicting anecdotal claims that it delays recovery. The free-range housing also had positive effects on appetite and recovery of energy balance.

## 1. Introduction

Many horses are currently housed in individual box stalls in stables [1,2,3,4]. Box stalls facilitate supervision, individual feeding and grooming of the horses, but obviously limit their scope for free movement, exploration and social interaction. An alternative is a modern housing system where horses are kept in groups in paddocks with shelters and lying areas and with individual feeding controlled by transponders. Anecdotal information indicates concerns among sports horse trainers that lack of rest in such systems delays recovery and impairs competition performance. However, unpublished data [5] indicate that picky-eater Standardbred trotters kept in a group housing system have better body condition than when housed in box stalls, indicating better appetite and higher feed intake. Environmental factors such as space allowance, group size and feeder characteristics have been shown to affect feed intake in pigs [6]. Little is known about how physical environment affects feed intake in horses. Keeping horses in groups may affect eating; which has been shown to be highly synchronized in group-housed horses [7,8]. 

In the short term, recovery involves decreasing muscle temperature, compensating for oxygen debt, and regulating acid–base balance. In the longer term, it also involves energy replenishment, fluid balance recovery, and tissue re-synthesis. Energy balance can be monitored by measuring body weight, energy expenditure (using heart rate), body condition score (BCS), and substrate usage (non-esterified fatty acids (NEFA) and urea). NEFA, originating from lipolysis of adipose tissue, have been widely shown to increase in ponies and horses during periods of insufficient energy intake [9,10,11,12,13]. When amino acids are used as energy substrate there is a degradation that starts with deamination, where the amino group is removed and converted into ammonia. Ammonia released by this process is removed from the body by forming urea in the liver. 

This study examined whether recovery of energy balance after competition-like exercise in Standardbred horses fed ad libitum was affected by housing system. Two different systems were compared: free-range group housing (FreeR) and an individual box-stall housing system (BOX) in which activity in groups was possible for only 4–5 h/day. The hypothesis was that free-range housing hastens recovery compared with box stall housing.

## 2. Materials and Methods

Umeå local ethics committee approved the study (**A 54-13**) and it was performed in compliance with European Union directives on animal experiments (2010/63/EU; European Union, 2010) and with laws (Swedish Constitution, 1988:534) and regulations (Swedish Board of Agriculture Constitution, 2012:26) governing experiments on live animals in Sweden.

### 2.1. Horses and Management

Eight adult Standardbred geldings in training (mean age 11 years, range 9–13 years) were used. The study was performed during May–June 2015 and all horses had raced during the period 2005–2015 and had average earnings of 146,553 SEK (range 26,500–495,798 SEK). Mean bodyweight at the beginning of the trial was 509 kg (range 410–562 kg). The horses were trained under the supervision of professional trainers (licensed by the Swedish Trotting Association) according to a training program similar to that used by Swedish trotting trainers [14]. 

### 2.2. Experimental Design

The horses were randomly allocated to two groups of four and kept in the box stall (BOX) or free-range group housing system (FreeR) for 21 days, followed by a complete change-over to the other treatment. Between treatments, the horses had a three-day transition period in the new housing system. All horses performed two similar exercise tests (ET), on day 7 (ET1) and day 14 (ET2) in each treatment period. On days 3, 11, and 18 of each treatment, the horses were exercised on a track (5000 m warm-up, 3000 m at a speed of 11.1 m/s).

The horses in treatment BOX were housed individually in 3 m × 3 m boxes with wood shavings and were let out into a sand paddock (2000 m^2^) together with the other horses in their group for 4–5 h every day. In the boxes, the horses had ad libitum access to water in buckets. The horses in treatment FreeR were kept together in a group housing area that consisted of a paved paddock (3200 m^2^) with a shelter with rubber matting and automatic feeding stations. They were offered water ad libitum in water barrels.

### 2.3. Diet

In treatment BOX, the horses were fed forage ad libitum in their stall, but no feed was offered during the paddock stay. Feed was offered four times per day (07.00, 12.30, 17.00, and 20.00 h) and there had to be left-overs at every meal to provide ad libitum access. 

In treatment FreeR, the horses were fed from automatic feeding stations that recognized the individuals by transponders. With this technique, feed allowance was regulated by time and all horses had access to the feeding station for more time than they used it (free access). All horses but one had 400 min eating time/day, while in one horse the access was 500 min/day to ensure free access. 

The same forage was used in both housing systems, a haylage (dry matter (DM) 78%, 11.2 MJ metabolizable energy (ME)/kg DM, 14.3% crude protein (CP), calcium (Ca) 5.6 g/kg DM, phosphorus (P) 2.2 g/kg DM, and magnesium (Mg) 1.7 g/kg DM). The horses were also given 0.5 kg concentrate (Krafft Sport, Malmö, Sweden (12 MJ ME/kg, 11% CP)) and 80 g mineral and vitamin supplement (Krafft Miner Vit, Malmö, Sweden) (Ca 55 g/kg, P 65 g/kg, Mg 60 g/kg, salt (NaCl) 125 g/kg, copper (Cu) 900 mg/kg, selenium (Se) 15 mg/kg, vitamin A 100,000 IU/kg, vitamin D3 10,000 IU/kg, and vitamin E 5000 mg/kg) every day. In the BOX system this was fed once a day, while in FreeR the automatic feed station divided the total allowance equally between every hour of the day. 

The horses were offered ad libitum access to salt blocks during the whole study and they also had extra salt mixed with beet pulp after the exercise on days 3, 11, and 18 (250 g beet pulp and 20 g NaCl).

### 2.4. Exercise Test

On the day of the exercise test (ET), the horses were transported 50 km to an official 1000-m oval, banked, gravel racetrack (Östersunds race track, Sweden). The horses performed the ET in groups of four, two from each treatment. The same driver drove the same horse in a harness race sulky on all test occasions and the horses raced in the same group at the same time on all occasions. The ET started with a warm-up consisting of 4000 m slow trot (6.3–6.7 m/s) and 500 m trot (10 m/s). After the warm-up, the horses walked the track for 10 min. They then trotted 2140 m in the same race field position, at 11.8–12.2 m/s for the first 1640 m and as fast as they could (free positioning) for the last 500 m. This was followed by a cool-down of 1000 m slow trot (6.3–6.7 m/s). Approximately one hour after crossing the finish line in the ET, the horses were transported back home, and three hours after the ET they were put back into their housing system.

### 2.5. Measurements, Sampling, and Analysis

The horses were weighed on a scale (Tru-test E2000S2, Auckland, New Zealand) every morning. Body weight measurements on the day before ET day and on the morning of ET day were used as “Rest” values for bodyweight. Rectal temperature was measured every day, at 07.00 h. Before and after each experimental period, the body condition score (BCS) of all horses was evaluated as specified by Henneke et al. [15]. During days 15–22 (in period 1) and days 14–19 (in period 2), forage intake was measured by weighing the allowance and left-overs. In treatment FreeR, the horses were fed forage from two feeding stations (four feeding places) and intake had to be measured for the whole group. Mean ambient temperature on ET days and the three following days was 8.4 °C (range 1.3–9.8 °C).

During ET days, blood samples were collected via a catheter inserted in vena jugularis after local cutaneous anesthesia (Tapin (lidocaine 25 mg/g, prilocaine 25 mg/g), Orifarm Generics, Stockholm, Sweden). The catheter was inserted approximately one hour before the first blood sampling. Blood samples were collected in 6-mL lithium-heparinized tubes (102 IU) and kept on ice until centrifuging (10 min, 920 × g, 18 °C), after which the plasma was frozen (−20 °C). Blood samples were collected at rest in the stable (Rest), at the racetrack 1 min after the finish line (FL) and after 10, 180, 240, 300, 360, and 420 min of recovery. At 20 and 44 h after the ET, blood samples were collected by venipuncture from the jugular vein. 

Total plasma protein concentration (TPP) was measured in all plasma samples using a handheld refractometer (Atago, Tokyo, Japan). Plasma lactate concentration was analyzed in samples Rest, FL and after 10, 180, and 420 min of recovery, using an enzymatic (L-lactate dehydrogenase and glutamate-pyruvate transaminase) and spectrophotometric method (Boehringer Mannheim/R-Biopharm, Darmstadt, Germany), with intraassay coefficient of variation (CV) 2.2% in this study. Plasma NEFA concentration was analyzed in samples taken at Rest, FL, and after 10, 180, 240, and 420 min of recovery and also after 20 and 44 h, by quantitative determination using an enzymatic colorimetric method (Wako Chemicals GmbH, Neuss, Germany), with intraassay CV 1.8% in this study. Plasma urea concentration was analyzed with a spectrophotometric method (Urea Assay Kit, Cell Biolabs Inc., San Diego, CA, USA), with intraassay CV 1.3% in this study. 

Heart rate was continuously recorded during the race and up to 420 min of recovery using a heart rate recorder (Polar CS600X Polar Electro, Kempele, Finland) and the data were analyzed using Polar ProTrainer 5 Equine Edition Software (Polar Electro, Kempele, Finland). Mean recovery heart rate was calculated using recordings from 270–410 min of recovery. 

### 2.6. Statistical Analysis

Analysis of variance was performed with the MIXED procedure in SAS (version 9.4; SAS Institute Inc., Cary, NC, USA), using an autoregressive (AR(1)) structure. Plasma sample results were pooled into recovery 3–7 h (180–420 min), and recovery 20–44 h (22 h and 44 h). Statistical analysis was performed with a statistical model including fixed effects of housing, sample, and the interaction between them. The model for an observed variable of horse i in housing j, sample k, was:

Y_ijk_ = μ + η_i_ + π_j_ + γ_k_ + (πγ)_ik_ + e_ijk_, (1)

where μ is the overall mean, η_i_ is the effect of horse, π_j_ is the effect of housing, γ_k_ is the effect of sample, (πγ)_ik_ is the effect of the interaction between housing and sample, and e_ijk_ is the random error. The random part included horse, horse × housing, and period. Observations within each horse × period × housing combination were modeled as repeated measurements. For urea, race had a significant effect and was included in the model.

Forage intake, bodyweight, BCS, heart rate, and velocity data were analyzed by a statistical model including fixed effects (housing, period, and the interaction between housing and period). The model for an observed variable of horse i in period j, in housing k was:

Y_ijk_ = μ + η_i_ + π_j_ + γ_k_ + (πγ)_ik_ + e_ijk_, (2)

where μ is the overall mean, η_i_ is the effect of horse, π_j_ is the effect of housing, γ_k_ is the effect of sample, (πγ)_ik_ is the effect of the interaction between housing and sample, and e_ijk_ is the random error. The random part included horse and horse × housing. Observations within each horse were modeled as repeated measurements. 

Post-hoc comparisons were adjusted for multiplicity using the Bonferroni method. Values are presented as least square means (LSM) with the standard error of the mean (SEM) in a parenthesis. Differences were considered statistically significant at *p* < 0.05.

## 3. Results

One of the horses was excluded due to a hoof crack, although it showed no clinical signs of pain. Training and ET were still performed with that horse and it was included in feed intake measurements, since these were done by group. Group forage intake was higher in treatment FreeR than treatment BOX (FreeR: 48 (1.7), BOX: 39 (1.7) kg (*p* = 0.003)) and the mean bodyweight during the whole treatment periods (21 days) tended to be higher in FreeR horses than in BOX horses (FreeR: 505 (13), BOX: 500 (13) kg (*p* = 0.07)). There was no difference in BCS between the housing systems (FreeR: 4.8 (0.4), BOX: 4.7 (0.4) (*p* = 0.93)). 

### 3.1. Rest 

Body weight was higher in FreeR horses than BOX horses at Rest (morning day before ET + morning before ET) (Table 1). Housing did not affect plasma NEFA, urea, or TPP concentration on the morning before ET (Table 1 and Table 2).

### 3.2. Finish Line and 10 min Recovery 

Housing system did not affect peak heart rate (FreeR: 224 (2), BOX: 221 (2) beats/min), plasma lactate concentration (Table 2), or velocity during the simulated race (FreeR: 12.0 (0.1), BOX: 12.0 (0.1) m/s). 

Plasma lactate concentrations and TPP were higher than Rest values at the finish line and after 10 min of recovery during both FreeR and BOX (Table 1 and Table 2). Plasma NEFA concentrations were higher than Rest values at 10 min of recovery during both FreeR and BOX, and for BOX treatment also at the finish line (Table 2). Plasma urea concentrations were higher than Rest values at the finish line (tendency *p* = 0.071) and after 10 min of recovery during BOX treatment (Table 2). There was no effect of FreeR compared with BOX on plasma NEFA, urea, or TPP concentration at finish line or after 10 min of recovery (Table 1 and Table 2). 

### 3.3. Short-Term Recovery (3–7 h)

**Figure 1:**
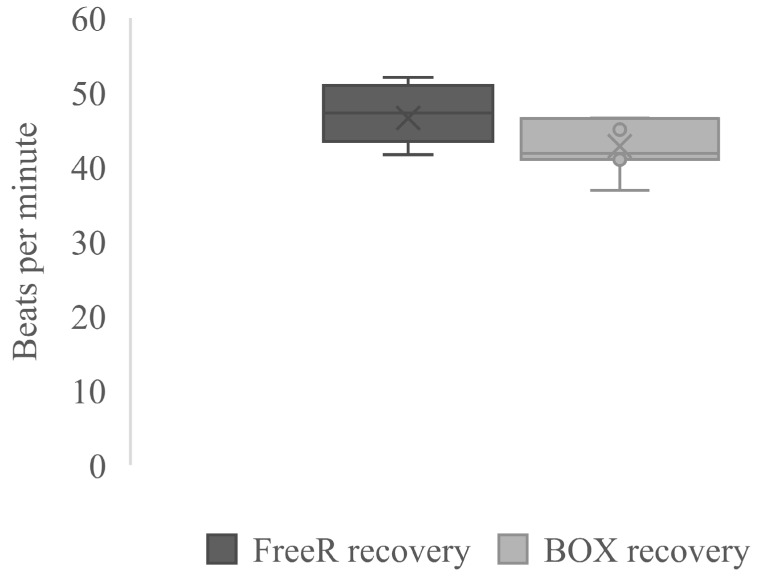
Box-plot of 3–7 h recovery heart rates in Standardbred horses kept in free-range group housing (FreeR) or box housing (BOX).

As mentioned, heart rate, plasma lactate, NEFA, urea, and TPP concentrations all increased from rest to race, and therefore recovery was necessary. There was a tendency during 3–7 h recovery for higher heart rate in FreeR horses compared with BOX horses (47 (1) vs. 43 (2) beats/min; *p* = 0.100) (Figure 1). The different housing systems did not significantly affect plasma lactate, plasma NEFA, urea, or TPP concentration during 3–7 h recovery (Table 1 and Table 2). During short-term recovery, plasma urea concentrations were greater than the Rest values (Table 2). During short-term recovery, TPP were higher than the Rest values during BOX housing (Table 1).

### 3.4. Long-Term Recovery (20–44 h)

Body weight did not return to the Rest values during long-term recovery (Table 1). Plasma NEFA returned to the Rest value during 20–44 h of recovery in BOX horses, while in FreeR horses it not only returned to the Rest value but fell below it (Table 2). Plasma NEFA was lower in FreeR horses than in BOX horses during 20–44 h of recovery (Table 2) and bodyweight was higher in FreeR horses than in BOX horses during this period (Table 1). Housing system had no significant effect on plasma urea or TPP concentration during 20–44 h of recovery (Table 1 and Table 2). Plasma TPP concentration during 20–44 h of recovery was significantly higher than the Rest value (Table 1). 

## 4. Discussion

In this study, there was little or no difference in short-term (3–7 h) metabolic recovery in horses kept in the free-range and box stall housing systems evaluated. In fact, the results indicated that in the long-term perspective, the free-range system may be beneficial. The lack of differences in NEFA, urea, and TPP responses during short-term recovery indicates that water and feed intake were similar in the two housing systems during the first hours after ET. During long-term recovery, the NEFA levels were very low in FreeR horses, even lower than before the ET. This indicates quick and efficient recovery of energy balance by horses in this housing system, an indication supported by the higher daily feed consumption observed in the FreeR housing system. The hormone insulin plays an important role in regulating lipolysis in adipose tissue, as increased insulin concentration results in decreased lipolysis and thereby decreased release of NEFA. An increased insulin response in FreeR (due to higher feed intake) may have lowered the NEFA response but the response may also have been influenced by different feeding intervals in the housing systems. Low post-exercise appetite has been observed in athletic horses (box-housed) and is suggested to be associated with the hormone’s active ghrelin, adiponectin, and leptin, and/or gastric ulcers [16,17]. Low BCS and periods of low appetite are conditions seen in some horses during periods of intense training and racing, and our results indicate that a group housing system might counteract these problems. Our findings indicate that physical environment is important for feed intake in horses, as reported earlier for pigs [6]. The lower feed intake in box-housed horses could have been due to the daily 4–5 h without feed in the paddock, but that is close to the time span without eating observed in wild-living horses and was probably compensated for by increased feed intake. Compensatory increases in feed consumption after a period of feed restriction have been reported in ponies [18]. 

Although plasma NEFA concentrations indicated that energy balance was restored in the horses, bodyweight was still not back to resting values at 44 h of recovery. In French Trotters, the post racing decrease in bodyweight is reported to be on average 9.4 kg (range 0–26 kg) and bodyweight requires on average 3.3 days to recover [19]. An exercise-induced decrease in body weight also seems to be affected by whether the horses are transported [20]. It has been suggested that sweat losses are responsible for 90% of post-exercise weight losses [21], which are therefore not affected by energy balance but by water and electrolyte intake. Since total plasma protein concentrations remained elevated from resting values at the end of the study period, it is likely that the horses did not recover fluid balance within 44 h. The salt intake in the present study might have been too low to compensate for losses, since salt blocks have been shown to be an inadequate sole source of salt for athletic horses [22] and the extra salt offered (20 g) to the horses on the day after ET might not have been sufficient.

There were no differences in plasma lactate responses between the housing systems, which was probably due to events in the immediate recovery period after exercise (i.e., 1000 m slow-down trot, walk, and road transport back home) being the same in both treatments. It has been shown previously that, during the first 30 min after intense exercise, lactate removal can be increased two-fold by light exercise [23]. Interestingly, there was no significant difference in HR between the housing treatments during short-term recovery indicating that energy expenditure was similar in this period [24]. In both systems, HR seemed to be slightly higher than earlier observed [25] in box-stalled Standardbred horses at night (36–40 bpm vs. 43–47 bpm in our study). We expected HR to be higher when horses were kept in group housing compared to box housing, due to more physical activity (walking around). Earlier studies have shown that physical activity in stabled or partly stabled horses is lower than in horses housed in free-range systems [26]. In addition, keeping horses in groups in paddocks seems to increase physical activity compared with keeping them in individual paddocks [27]. In BOX horses, an elevation of HR from expected levels at rest is difficult to explain and accordingly also the lack of difference in HR between treatments. One possible reason for the lack of difference in HR is because HR was elevated in BOX horses due to horses being more alert, excited, or stressed in this system (by people and horses moving around in the stable). This assumption contradicts anecdotal claims that horses kept in a free-range group housing system are less relaxed than stabled horses. 

## 5. Conclusions

This study found that a free-range housing system hastened recovery in Standardbred trotters, rather than delaying it as suggested by anecdotal claims. The free-range housing system also had positive effects on appetite and recovery of energy balance. 

## Figures and Tables

**Table 1:** Body weight and total plasma protein in Standardbred horses kept in free-range group housing (FreeR) or box housing (BOX). SEM = standard error of the mean.

Variable	Sample	FreeR	SEM	BOX	SEM	*p*-Value
Body weight (kg)						
	Rest	509	13	504	13	0.038
	20−44 h recovery	504 ^a^	13	498 ^a^	13	0.020
Total plasma protein (g/L)						
	Rest	59.5	0.8	60.4	0.8	0.996
	Finish line	73.5 ^a^	0.8	74.3 ^a^	0.8	1.000
	10 min recovery	67.0 ^a^	0.8	67.4 ^a^	0.8	1.000
	3−7 h recovery	61.0	0.8	62.3 ^a^	0.8	0.405
	20−44 h recovery	61.8 ^a^	0.8	62.6 ^a^	0.8	1.000

^a^ Significantly different (*p* < 0.05) from Rest.

**Table 2:** Plasma concentrations of non-esterified fatty acids (NEFA), urea and lactate in Standardbred horses kept in free-range group housing (FreeR) or box housing (BOX). SEM = standard error of the mean.

Variable	Sample	FreeR	SEM	BOX	SEM	*p*-Value
Plasma NEFA (mmol/L)						
	Rest	0.26	0.04	0.21	0.04	1.000
	Finish line	0.33	0.04	0.34 ^a^	0.04	1.000
	10 min recovery	0.54 ^a^	0.04	0.55 ^a^	0.04	1.000
	3–7 h recovery	0.24	0.04	0.22	0.04	1.000
	20–44 h recovery	0.16 ^a^	0.04	0.25	0.04	0.019
Plasma urea (mmol/L)						
	Rest	5.0	0.3	4.6	0.3	0.481
	Finish line	5.1	0.3	4.9 ^b^	0.3	1.000
	10 min recovery	5.2	0.3	4.9 ^a^	0.3	0.818
	3–7 h recovery	5.6 ^a^	0.3	5.3 ^a^	0.3	0.718
	20–44 h recovery	4.8	0.3	4.7	0.3	1.000
Plasma lactate (mmol/L)						
	Rest	0.9	0.8	1.3	0.8	1.000
	Finish line	20.8 ^a^	0.8	20.6 ^a^	0.8	1.000
	10 min recovery	16.0 ^a^	0.8	16.0 ^a^	0.8	1.000
	3–7 h recovery	1.0	0.7	1.1	0.7	1.000

^a^ Significantly different (*p* < 0.05) from Rest. ^b^ Tendency for significant difference (*p* ≤ 0.09) from Rest.
